# How a Lateralized Brain Supports Symmetrical Bimanual Tasks

**DOI:** 10.1371/journal.pbio.0040158

**Published:** 2006-05-09

**Authors:** Roland S Johansson, Anna Theorin, Göran Westling, Mikael Andersson, Yukari Ohki, Lars Nyberg

**Affiliations:** **1**Department of Integrative Medical Biology, Physiology Section, Umeå University, Umeå, Sweden; **2**Umeå Center for Functional Brain Imaging, Umeå University, Umeå, Sweden; **3**Department of Psychology, Umeå University, Umeå, Sweden; University of MinnesotaUnited States of America

## Abstract

A large repertoire of natural object manipulation tasks require precisely coupled symmetrical opposing forces by both hands on a single object. We asked how the lateralized brain handles this basic problem of spatial and temporal coordination. We show that the brain consistently appoints one of the hands as prime actor while the other assists, but the choice of acting hand is flexible. When study participants control a cursor by manipulating a tool held freely between the hands, the left hand becomes prime actor if the cursor moves directionally with the left-hand forces, whereas the right hand primarily acts if it moves with the opposing right-hand forces. In neurophysiological (electromyography, transcranial magnetic brain stimulation) and functional magnetic resonance brain imaging experiments we demonstrate that changes in hand assignment parallels a midline shift of lateralized activity in distal hand muscles, corticospinal pathways, and primary sensorimotor and cerebellar cortical areas. We conclude that the two hands can readily exchange roles as dominant actor in bimanual tasks. Spatial relationships between hand forces and goal motions determine hand assignments rather than habitual handedness. Finally, flexible role assignment of the hands is manifest at multiple levels of the motor system, from cortical regions all the way down to particular muscles.

## Abstract 2

Johansson and colleagues combine the use of a novel bimanual task with neurophysiological and brain imaging experiments to examine the neural basis and dynamics of flexible hand coordination during object manipulation.

## Introduction

A hallmark of human behavior is the ability to coordinate the two hands for effective manipulation of the environment. Bimanual coordination has been the subject of intensive investigation the last decades. Most of this research has concerned situations in which the two hands have difficulties acting independently [ 1, 2], rather than on how they are coordinated for common object-oriented goals as in natural manipulations [ 3]. Studies that address object manipulations have principally dealt with tasks characterized by different and asymmetric engagement of the two hands [ 4– 13]. A large repertoire of bimanual manipulations, however, involves symmetric forces of the two hands. In particular, whenever we grasp bimanually a single object, whether opening a jar, bending a rod or molding a snowball, the hands must be able to orchestrate equal and opposing forces. In this paper, we ask how the functionally lateralized brain handles this basic problem of spatial and temporal complexities. A fundamental question is whether the brain allocates different functional roles to the hands as previously suggested for many bimanual skills, with one hand primarily acting and the other stabilizing the object as to provide frames of reference for the acting hand [ 5, 6, 9]. If so, one possibility is that habitual handedness determines acting hand, making the right hand prime actor for right-handed persons. Indeed, handedness is the most recognized human behavioral asymmetry and links to cerebral hemispheric differences [ 14– 16].

An alternative possibility is that the functional roles of the hands are flexible and can change across tasks and task phases. Such flexibility could relate to how bimanual actions are conceptualized with reference to their spatial goals, or more specifically, to the mapping between hand forces and desired movement outcomes. For example, when removing a stopper from a bottle held by one hand, the other hand grasping the stopper, be it left or right depending on overall task constraints, may be appointed as prime actor because the forces it generates are aligned with the goal motion, that is, to pull the stopper out of the bottle. However, if the task instead would entail moving the bottle rather than the stopper, as might occur in rare instances, whichever hand that grasps the bottle may be prime actor. Likewise, when we open or close a jar by generating bimanual twist forces, the hand holding the lid would be the prime actor as long as the intention is to rotate the lid rather than the jar. By this view, the brain assigns as prime actor the hand (right or left) whose forces are spatially congruent with the movement goal while the accompanying hand, bound to generate forces directed opposite to the goal motion, is assigned an assisting, stabilizing function.

We addressed critically these alternatives in a task where study participants applied light isometric forces to a rigid unsupported tool (weighing 150 g) held between the two hands in order to control a cursor on a screen ( Figure 1). The task was to hit successively displayed visual targets as quickly as possible. Compressing and stretching forces applied between the handles of the tool along its longitudinal axis moved the cursor horizontally and twist forces (torques) applied around this axis moved the cursor vertically ( Figure 1A). Participants had to generate various combinations of longitudinal and twist forces to hit the target, since it reappeared at an unpredictable location on the screen after each hit. Note that, any force generated by one hand must be counterbalanced by opposing forces generated by the other hand because the tool was freely held in the air.

**Figure 1:**
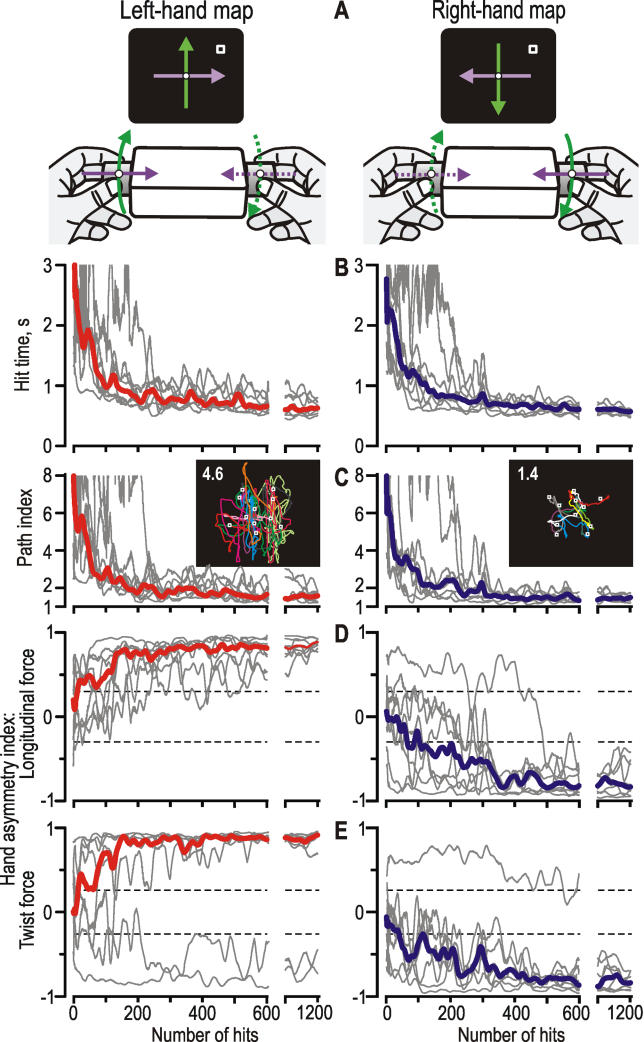
The Bimanual Target-Chasing Task and Flexible Role Assignment of the Hands (A) Mappings between applied forces and cursor movements (top graph). With the left-hand map, the cursor moved horizontally in the direction of the longitudinal force applied by the left hand (solid purple arrows). A counter-clockwise twist force applied between the handles (as if unscrewing the lid of a jar) moved the cursor upward (solid green arrows). With the right-hand map the cursor moved in the opposite directions and thus in the direction of the forces of the right hand. (B and C) Performance under each mapping rule shown for a complete first session with 602 hits and from the last 100 hits of a second session. Superimposed thin lines show hit time and path index for each participant as a function of target number (data median-filtered over a ± 10-s period around each hit). Solid curve give medians across participants. Inserts in (C) exemplify cursor trajectories with a median path index of 4.6 and 1.4 across 10 target transitions. The targets, distributed about uniformly over the screen, were located 5.1 ± 2.1° (mean ± 1 SD) visual angle from its center, which corresponded to 2.2 ± 0.3 N force applied tangentially to the surfaces of the handles. (D and E) Hand-asymmetry indices computed for a sliding ± 10-s time window. Horizontal lines give the upper and lower 95% confidence limit of the index, postulating that hand selection would have occurred randomly. A significant positive and negative index indicates left and right-hand primarily acting, respectively.

To assess whether spatial congruency between hand actions and goal motions might influence possible functional role differentiation between the hands, each participant experienced two different mapping rules relating bimanual forces and cursor movements, one congruent with the left hand and one with the right hand. That is, with the “left-hand map,” the cursor moved in the direction of the forces applied by the left hand and consequently in a direction opposite to the forces applied by the right hand (left panel in Figure 1A). Hence, longitudinal compression forces moved the cursor to the right side of the screen and stretch forces to the left side while counterclockwise twist forces moved the cursor to the upper part of the screen and clockwise forces to its lower part. The “right-hand map” was reversed compared to the left-hand map and the cursor thus moved with the forces of the right hand and opposite to those of the left hand (right panel in Figure 1A). We report that the brain flexibly appoints one hand as prime actor even though the task required that the hands generated symmetrical forces. Furthermore, the choice of acting hand depended on the mapping rule and correlated with lateralized activity in distal hand muscles, corticospinal pathways, and primary sensorimotor and cerebellar cortical areas. We also report on lateralized engagement of cortical premotor areas in the task.

## Results

### Behavioral Asymmetries of the Hands

In a first experimental series, we examined behavioral asymmetries between the hands. We reasoned that if one of the hands primarily acted, the tool would tend to move in the direction of the forces generated by this hand because it would receive stronger motor commands or commands that would precede those of the accompanying hand [ 17, 18]. We indeed observed both lateral and rotational tool movements, confirming that the motor commands of the two hands were asymmetric. These movements were, however, small and for a typical cursor transition between successive targets the lateral movement was some 2 mm and the rotational one around 2° ( Figure 2). To establish measurements of hand asymmetry we correlated the movements recorded with hand forces. One hand-asymmetry index was calculated for longitudinal forces and one for twist forces, by computing the correlation between longitudinal forces and lateral tool movements, and between twist forces and tool rotations, respectively ( Figure 2).

**Figure 2:**
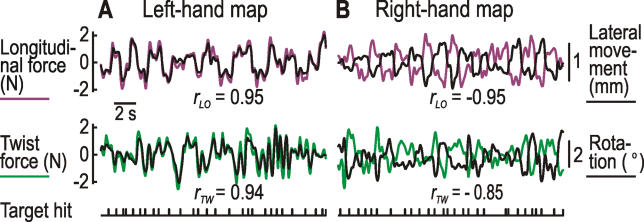
Influence of Mapping Rule on Tool Movements during Performance with Left-Hand and Right-Hand Maps Superimposed time traces of longitudinal force and lateral tool movement (upper panels) and of twist force and rotational tool movement (lower panels) from a single participant during the last 20 s of target chasing in the first experiment; *r _LO_* and *r _TW_* indicate hand-asymmetry indices for longitudinal and twist forces. Bottom trace represents instances of target hits (spikes). For the last 20 s of runs by all participants and mapping rules, the slope coefficients of the linear regressions indicated that the tool moved 0.84 (0.28–1.23) mm/N longitudinal force and rotated 0.84 (0.36–1.42) °/N twist force (median and 25th–75th percentile). The corresponding values for the 30-s periods of target chasing for which fMRI data were analyzed (see Figure 5A and 5B), where the wrists of the participants were strapped, were 0.28 (0.19–0.68) mm/N and 0.63 (0.42–1.04) °/N.

**Table 1:**
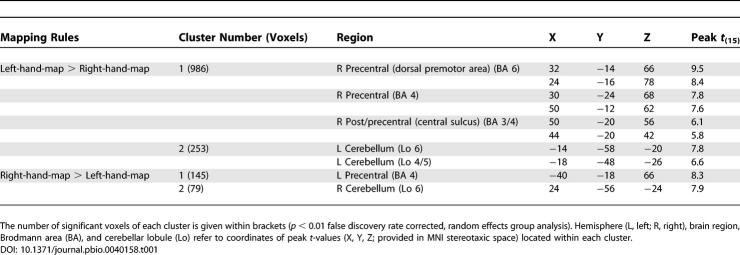
Areas with Different BOLD Responses during Target Chasing under the Left-Hand and Right-Hand Mapping Rules

Reliable hand asymmetries developed while participants ( *n* = 8) gradually learned the task. With either mapping rule it took some 300 target hits before they approached a rather stable performance as measured by the time between sequential target hits (“hit time”; Figure 1B) and by a cursor path index pertaining to the straightness of the cursor trajectories between successive targets ( Figure 1C) (see further [ 19]). The path index was the ratio between the distance the cursor traveled between hits and the actual inter-target distance. During learning, as shown by a decline in hit time and path index, the hand-asymmetry indices gradually approached 1 or −1 in all participants ( Figure 1D and 1E), which indicated that the tool consistently moved either with the forces applied by the left or by the right hand, respectively. Furthermore, the hand-asymmetry index for longitudinal forces indicated that the tool moved for all participants with the direction of the left hand force with the left-hand map and with the right hand force with the right-hand map ( Figure 1D). Thus, spatial congruency between hand forces and cursor movements determined acting hand. For twist forces, at the end of the second session all participants appointed the right hand as prime actor with the right-hand map and all but two the left hand with the left-hand map ( Figure 1E).

### Map-Dependent Asymmetric Use of Hand Muscles

The map-dependent asymmetric behavior of the hands indicated that the mapping rule influenced the motor commands of hand muscles. Using surface electromyography (EMG), we examined this issue in eight well-practiced participants. According to the hand-asymmetry indices, for all participants the acting hand matched the prevailing nominal mapping rule for both longitudinal and twist forces. For each hand, we recorded from two distal (intrinsic) hand muscles operating on the index finger and thumb (first dorsal interosseous, 1DI; abductor pollicis brevis, APB) and from two proximal (extrinsic) hand muscles in the forearm operating on the wrist (extensor carpi radialis, ECR; flexor carpi ulnaris, FCU). To obtain data pertaining to discrete manual action components, we distributed the targets such that the hands applied predominantly a longitudinal compression or stretch force, or a twist force corresponding to a supination or a pronation force. That is, every other target was in the center of the screen (zero force) and every second was located 4.5° above, below, to the right or to the left of the center target. Furthermore, we focused on hand actions that brought the cursor from the center target.

Despite that the hands generated practically identical forces with either mapping rule, it markedly influenced the EMG signals especially from the distal hand muscles ( Figure 3). For each pair of homonymous muscles we used a repeated measures analysis of variance (ANOVA) to assess effect of map, hand (left, right) and manual action component (compression, stretch, supination, pronation) on the relative change in EMG amplitude from the advent of a peripheral target to the instance of maximal force rate ( Figure 3C). As expected from anatomical relationships, the action component had a main effect for all muscles (F _(3,21)_ > 11.3 in all instances; *p* < 0.0002). For example, APB was more active in pronation than in supination, and 1DI and FCU were most active during stretch force and ECR during compression force. Most important, the map reliably influenced the use of APB (F _(1,7)_ = 62.3, *p* < 0.0001) and 1DI (F _(1,7)_ = 23.9, *p* < 0.002) whereas it had a weaker effect on the ECR (F _(1,7)_ = 8.15, *p* < 0.03) and no reliable effect on FCU (F _(1,7)_ = 2.55, *p* = 0.15). For each hand, a nominally congruent mapping between hand action and cursor movement rendered overall stronger activation of especially the distal muscles as compared to an incongruent mapping ( Figure 3C). Furthermore, the mapping rule and the manual action component interacted for the distal muscles (F _(3,21)_ = 11.8, *p* < 0.0001 for APB and F _(3,21)_ = 4.9, *p* < 0.01 for 1DI). In accord with their mechanical effects on the digits, when a hand was acting as compared to assisting, the use of APB and 1DI markedly increased during pronation and compression forces, respectively. The influence of the mapping rule on the use of distal hand muscles explains the asymmetric behavior of the hands: because of elastic properties of muscles, the weaker engagement of especially the intrinsic muscles of the assisting as compared to the acting hand resulted in a yield for the assisting hand that equalized the forces for the two hands. That is, when a hand primarily acted, it operated with higher stiffness than when assisting, apparently implemented by higher contraction levels in the muscles of the acting hand.

**Figure 3:**
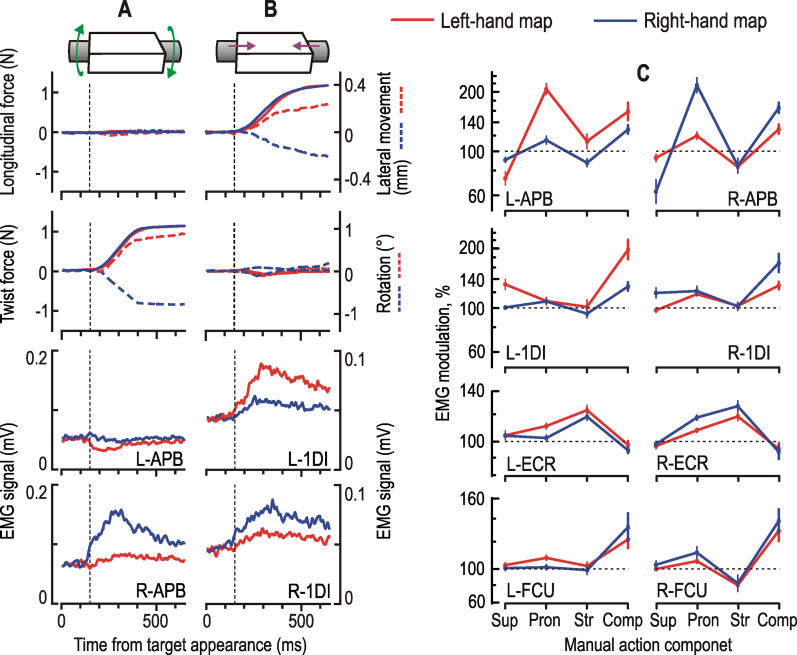
Effects of Mapping Rule on Use of Intrinsic (APB, 1DI) and Extrinsic (ECR, FCU) Hand Muscles (A) Solid curves and left abscissas show as a function of time longitudinal force, twist force and EMG signals from the left (L) and right (R) APB during vertical cursor movement generated by supination actions by the left hand together with pronation actions by the right (see top panel). Dashed curves show related tool movements (ordinate in [B]). Note the reversal in tool rotation with map changes (red and blue curves). (B) EMGs from the 1DI muscles during horizontal cursor movements generated by compression forces between the handles (ordinate in [A]). Note the reversal of lateral displacement associated with map changes. (A and B) Data averaged across all participants referenced to the appearance of the peripheral target. Force and object movement signals referenced to direction of supination and longitudinal compression by the left hand. (C) Relative change in EMG amplitude for all eight muscles during the center-out cursor movements plotted against manual action ( *Sup,* supination torque, *Pron,* pronation torque; *Str,* stretch force and *Comp*, compression force). Participant means (± 1SEM; *n* = 8) computed on a logarithmic scale. Irrespective of its location, the mean absolute value of the forces applied tangentially to the handles when hitting a peripheral target was 1.25 N.

### Map-Dependent Asymmetric Corticospinal Control

Since the corticospinal pathways play an important role for dexterous finger actions [ 20], having especially strong effects on distal hand muscles [ 20, 21], we asked whether the asymmetry in the control of hand muscles was reflected in the activity of these descending pathways. To estimate this activity we tested the excitability of the corticospinal systems using single-pulse transcranial magnetic brain stimulation (TMS) delivered to the primary motor cortices [ 22]. We recorded EMG potentials evoked in the muscles analyzed above in response to TMS pulses delivered 150 ms after a peripheral target first appeared, i.e., late during the reaction time of the muscle command that brought the cursor towards the target (dashed vertical lines in Figure 3A and 3B). For all muscles in either hand, TMS pulses delivered to the contralateral hemisphere rendered stronger EMG responses with the congruent than with the incongruent mapping between hand forces and cursor movements ( Figure 4). Stimulation of the ipsilateral hemisphere did not reliably evoke EMG responses (unpublished data). We quantified the excitability of the corticospinal system by analyzing the ratio between the peak amplitude of the evoked EMG responses and the corresponding background EMG activity measured in non-stimulated trials [ 22]. An ANOVA of the same design as described above showed that the mapping rule had a main effect on this ratio for all muscles (F _(1,7)_ ≥ 15.3; *p* < 0.006 in all instances) ( Figure 4B). Neither hand nor action component exerted a main effect. These findings indicate that the mapping rule influenced the activity of the corticospinal pathways and that pathways originating in the contralateral hemisphere were more active when a hand was acting as compared to assisting.

**Figure 4:**
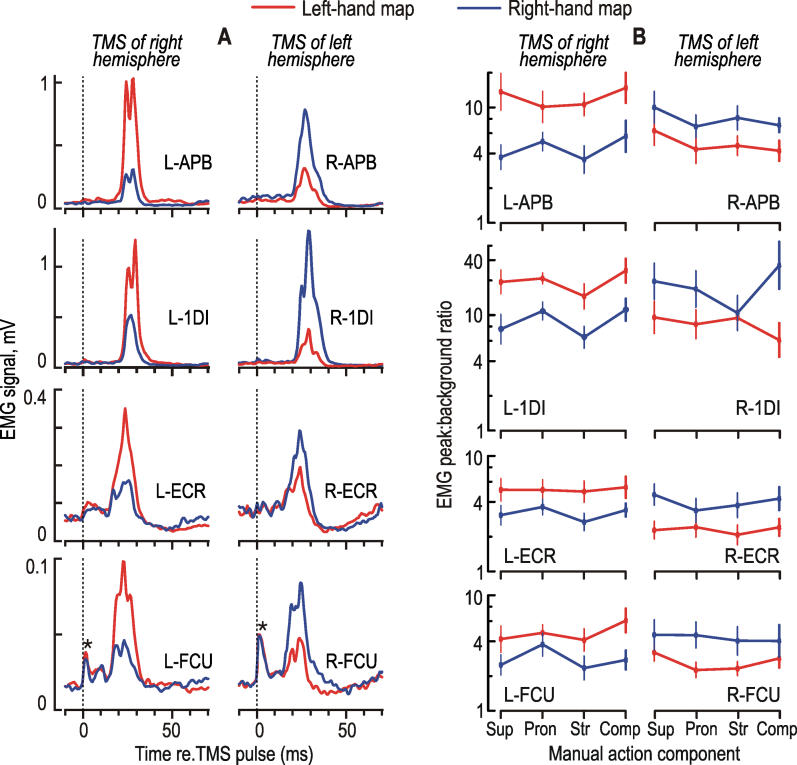
Effects of Mapping Rule on Excitability of Corticospinal Systems Originating in Each Hemisphere Assessed by TMS (A) Short latency EMG responses in intrinsic (APB, 1DI) and extrinsic (ECR, FCU) muscles of the left (L) and right (R) hand evoked by TMS delivered to the contralateral hemisphere in trials with primarily compression forces applied longitudinally to the tool. Data averaged across all participants referenced to time of the TMS pulse (dashed vertical line). Asterisks indicate stimulus artifacts. (B) Ratio between peak amplitude of evoked EMG responses and the corresponding background EMG activity measured in non-stimulated trials plotted against manual action component for all eight muscles. For further details, see Figure 3C.

**Figure 5:**
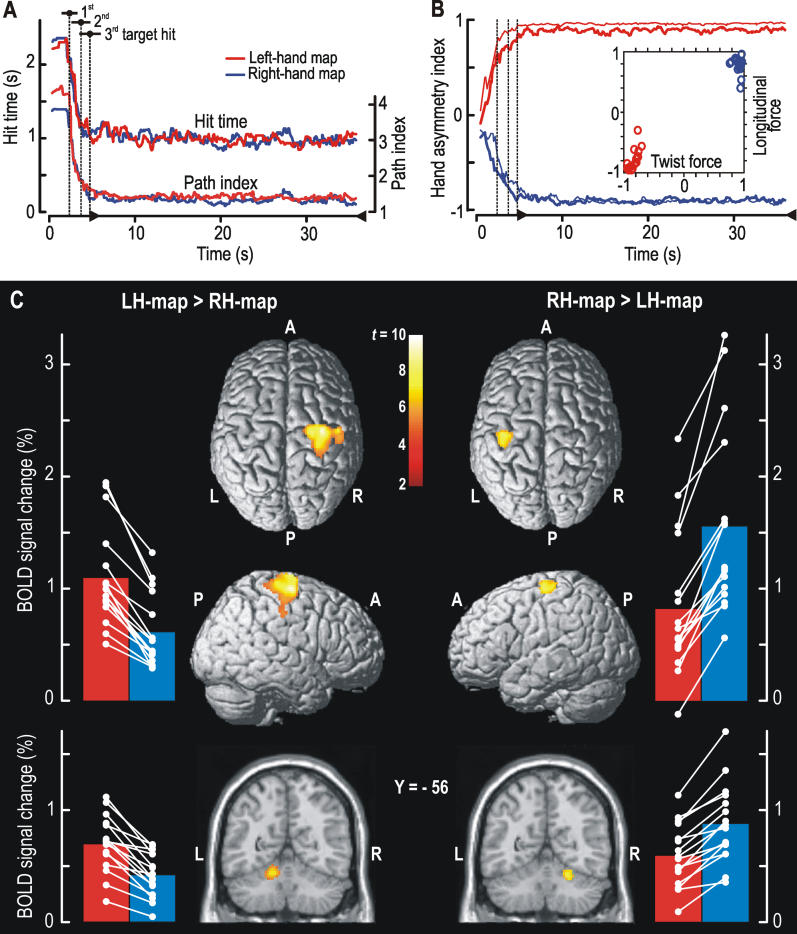
Brain Activity and Performance during Runs in the Scanner Averaged across Participants (A) Median hit time and path index as a function of time; arrowheads delimit the period used in fMRI analyses. Vertical lines indicate mean time for 1st, 2nd, and 3rd target hit with both maps (horizontal bar gives ± 1 SD). (B) Median hand-asymmetry index for horizontal (thick lines) and twist (thin lines) forces computed for a ±1-s sliding time window. Inset shows for each participant median indices for the delimited period plotted against each other. (C) Areas with stronger BOLD responses for left-hand map (LH-map > RH-map) and right-hand map (RH-map > LH-map). The surface rendered diagrams, based on single-participant standardized brain template in SPM2, do not indicate precisely locations of cortical activations since they may extend deep into sulci. See further Table 1. R, right; L, left; A, anterior; P, posterior. Top and bottom histograms give the percent BOLD signal change relative to mean of session as a function of mapping rule within the cortical and cerebellar cluster, respectively, for each contrast. Height of bars gives data averaged across participants and symbols joined by lines represent data from an individual participants.

### Map-Dependent Brain Activity

These neurophysiological results strongly suggested that the right and left hemispheres contributed differently to performance with the left-hand and right-hand map, respectively. We further investigated this issue with functional magnetic resonance brain imaging (fMRI) that measured changes in blood oxygen level-dependent (BOLD) signals, which correlate with changes in neural activity [ 23]. All participants ( *n* = 16) had practiced extensively with both maps before the fMRI scanning and the acting hand for both longitudinal and twist forces matched the nominal mapping rule.

In the scanner, each participant chased targets during four 36-s runs with each mapping rule ( Figure 5). The targets were located in all four quadrants of the screen with a distribution similar to that in the experiment focusing on behavioral analyses (see Figure 1C). Although we presented the maps in an order unpredictable to the participants, already after about two target hits these trained participants approached plateau performance for hit time and path index ( Figure 5A). Likewise, at this point the participants had assigned different roles for the hands depending on the mapping rule ( Figure 5B). Thus, in contrast to the gradual development of asymmetry of hand function in novice participants ( Figure 1B– 1E), skilled participants swiftly and flexibly switched acting hand when exposed to unpredictable alterations of the mapping rule. Because our focus here was on the steady-state adapted behavior, we analyzed fMRI images obtained during the last 30 s of the 36-s target-chasing runs. Neither the hit time nor the path index differed between the maps for this period ( *F*_(1,15)_ ≤ 0.10, *p* > 0.76 in either instance). For the hand-asymmetry index, a two-way ANOVA indicated a main effect of mapping rule ( *F*_(1,15)_ = 552.0, *p* < 10 ^−6^) but not of action component, i.e., longitudinal vs. twist force (inset in Figure 5B).

To reveal map-specific brain activity, in random effect group analyses we contrasted BOLD signals ( *p* < 0.01 corrected for false discovery rate) obtained with the left-hand map with those obtained with the right-hand map and vice versa ( Figure 5C and Table 1). The left-hand map specifically activated one cortical cluster with local maxima in the right primary sensorimotor and adjacent dorsal premotor areas and one in the junction between the anterior and posterior lobe of the left cerebellum. Conversely, the right-hand map activated a single cluster that included the left primary motor cortex and one in the posterior lobe of the right cerebellum. Thus, map change resulted in a between-hemisphere shift in the magnitude of activation of largely homologous primary cortical sensorimotor and cerebellar areas. Inspection of BOLD effect sizes indicated that both maps were associated with bilateral activity in all these areas, but the level of activation varied systematically as function of primarily acting hand ( Figure 5C).

The size of the sensorimotor cluster area in the right cerebral hemisphere was markedly larger than the corresponding cluster in the left hemisphere. In particular, the left-hand-map > right-hand-map contrast exposed dorsal premotor regions in the right precentral gyrus, whereas the reverse contrast did not expose corresponding premotor activity in the left hemisphere ( Figure 5C, Table 1). Asymmetric patterns of activation of motor cortex have been observed in many brain imaging studies comparing unimanual movements with the right and left hand, and it has been suggested that movements with the subdominant hand are more demanding with respect to the need of cortical control [ 24– 26]. An alternative explanation may be that either mapping rule activated premotor areas in the left hemisphere to a similar degree, complementing the notion that the left hemisphere in right-handers holds and instantiates critical components of sensorimotor engrams that underlie skilled actions irrespective of acting hand [ 14, 15, 24, 27– 29]. Additional analyses of the fMRI data supported the latter explanation. In a conjunction analysis that delineated cortical regions activated under both mapping rules ( *p* < 0.01 corrected for family-wise error [FWE] rate), we found common activations in left dorsal premotor cortex with local maxima both in its caudal and more rostral portions ( Figure 6: #2–#4) [ 30]. We also observed activation of the supplementary motor area with one maximum in its rostral portion (#5) and one in its caudal portion (#6), representing activation of pre-supplementary motor area and supplementary motor area proper, respectively [ 30]. Finally, one cluster with a single maximum was observed in a more ventral premotor area on the left inferior frontal gyrus (# 1). Inspection of BOLD effect sizes at local maxima verified that either mapping rule robustly activated these premotor areas (see histograms in Figure 6). Furthermore, with the exception for the most caudal dorsal premotor area that partly overlapped the cluster delineated by the right-hand-map > left-hand-map contrast ( Figure 6: # 4), the premotor areas identified in the left hemisphere were activated to a similar magnitude irrespective of mapping rule. Considering the right hemisphere, both mapping rules activated a single cluster that partly overlapped the region delineated by the left-hand-map > right-hand-map contrast, and this cluster included only one maximum that represented premotor activity ( Figure 6: #7). Taken together, these findings suggest that several premotor areas of the left hemisphere were activated to a similar degree irrespective of acting hand, whereas the activity of premotor areas of the right hemisphere depended more on hand selection.

**Figure 6:**
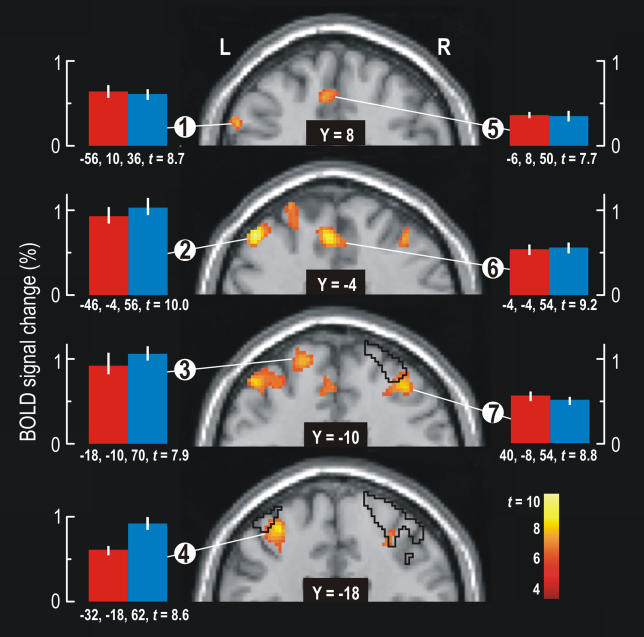
Premotor Cortical Areas with Increased BOLD Responses with Both the Left- and the Right-Hand Map Overlaid on Coronal Slices of the MNI T1-Weighted Brain Template For the left hemisphere (L), significant activations ( *p* < 0.01, FWE-corrected) occurred in one cluster (443 voxels) with two maxima in precentral gyrus (#2 and #4; BA 6), in one single-peak cluster (153 voxels) in superior frontal gyrus (#3; BA 6), in one cluster (281 voxels) with two maxima in medial frontal gyrus (#5 and #6; BA 6), and in one small single-peak cluster (29 voxels) in left inferior frontal gyrus (#1; BA 44). In the right hemisphere (R), there was one cluster (303 voxels) with three maxima, one of which was located in the precentral gyrus outside the cluster delineated by left-hand-map > right-hand-map contrast (#7; BA 6). Solid black lines in the left and right hemisphere outline the clusters identified with the right-hand-map > left-hand-map and left-hand-map > right-hand-map contrasts, respectively (see Figure 4C). Histograms give percent BOLD signal change relative to mean of session for the local maxima of identified clusters. Red and blue columns refer to left- and right-hand maps, respectively. Column height gives data averaged across participants and error bar ± 1SEM ( *n* = 16). Coordinates (X, Y, Z in MNI stereotaxic space) and *t*_(30)_ values for the maxima are presented below each histogram.

## Discussion

Our central finding is that the brain selects one hand as prime actor in bimanual object manipulations even in a task that necessitates symmetric forces by the two hands. Furthermore, the choice of acting hand is flexible and determined by spatial relationships between hand actions and goal motions. The hand that experiences the most natural directional relationship between forces generated and desired motion consequences, whether left or right, is appointed as prime actor while the accompanying hand plays an assisting, or postural, role. We have also shown that this asymmetric control of the hands is manifest at various levels of the motor system, from the cortical level all the way down to specific muscles. Thus, besides supporting the notion that there is a dual coordinated control scheme for goal oriented behaviors, with one system for goal motions and one for postural support [ 31], our findings suggest that these systems are segregated throughout the motor system and can be flexibly rearranged between the hands depending on task constrains. The system for goal motions is associated with increased cortical and corticospinal activity whereas the postural system appears hidden in these respects. Presumably, the goal system interacts primarily with exteroceptive sensory modalities (visual, auditory, and tactile) while proprioceptive and vestibular mechanisms may be most important for the postural system.

The obligatory appointment of one hand as prime actor apparently reflected a choice by the brain in implementing one of different optional spatial transformations that associate motor commands and goal motions, i.e., at least one distinct for each hand. Applying one of the possible transformations and suppression of others would prevent detrimental interference effects akin to those observed when participants attempt to execute simultaneously tasks thought to compete for partly the same neural resources [ 1, 32– 34]. Selection of the hand that offers the most spatially congruent action-perception relationship under prevailing task constraints would both optimize performance [ 35] and minimize computational demands [ 34, 36]. For twist forces, however, the selection of acting hand did not strictly conform to that expected from the nominal mapping rule (see the two exceptional participants in the left panel of Figure 1E). This behavior, which usually only applied to runs with one of the mapping rules for a given participant, presumably reflects a higher capacity of the brain to predict alternative spatial consequences of manual twist actions as compared to lateral actions that is rooted in daily experiences of object manipulations. For example, if holding a book horizontally between the hands, the visual consequences of a given pitch rotation (bimanual twist action) may be either an upward or a downward movement of the book depending on whether reference is to the near or remote edge of the book. In contrast, the predicted visual consequences of a lateral force are nearly always a directionally congruent sideways movement.

Our neurophysiological (EMG, TMS) and brain imaging (fMRI) results confirmed that the hand-asymmetry indices arise from a true functional hand asymmetry. That is, changes in acting hand as indicated by the hand-asymmetry indices affected multiple levels of the neuromuscular system compatible with a between-hemisphere shift of primary sensorimotor and cerebellar areas leading the task. Flexible switching of primarily acting hand not only occurs in tasks that involve symmetrical bimanual actions but certainly also in natural tasks with obvious role differentiation between the hands. For example, when a right-handed person fills up a glass from a decanter the right hand typically acts by picking up the flask in anticipation of performing the pouring act while the left hand is the prime actor in the bimanual sub-task of removing the stopper. We propose that flexible role assignment of the hands promotes effectiveness in the large variety of natural tasks that include sequences of subtasks across which the spatial mapping between actions and desired sensory goals vary between the hands. That is, when the precision requirements so allow, it reduces the need of reorienting and re-grasping objects as would be required if the habitually preferred hand always was selected as prime actor.

The observed left-lateralization of activity in premotor areas harmonizes with observations during unimanual tasks that suggest that motor areas of the left hemisphere provide critical ingredients of the executed sensorimotor programs irrespective of acting hand [ 15, 24, 28, 29]. Furthermore, the increased activation of premotor regions of the right hemisphere with the left-hand acting suggests important functions of premotor areas in the role appointment of the hands in bimanual tasks. We propose that left premotor areas during left-hand acting suspend the lead of left primary sensorimotor areas while simultaneously engaging premotor networks of the right hemisphere, thereby transposing the lead to right primary sensorimotor areas. There is evidence that connections of premotor areas through the anterior portion of the corpus callosum support intermanual coordination [ 37– 41]. Likewise, evidence in macaques suggest that arm selection in unimanual reaching engages dorsal premotor and supplementary motor areas [ 42, 43], presumably as a part of their roles in transforming movement parameters from an external visuospatial reference frame into a movement description [ 44– 47]. A pressing issue for future research is to unravel mechanisms and brain regions responsible for choice of primary actor in bimanual tasks. Hand selection could be a self-emergent property of the networks that embody the implemented action plan, or certain brain areas may be engaged transitorily to reconcile this selection.

## Materials and Methods

Thirty-seven neurologically normal right-handed [ 48] participants gave written informed consent to participate in this study, which was approved by the local ethical committee. Before the experiment, the participants were informed that forces applied longitudinally between the handles moved the cursor horizontally and twist forces vertically. They received no further instructions about the mapping rule or any suggestions about which hand to use preferentially. Before each experimental session, the participants were encouraged to hit the upcoming targets as fast as possible. The cursor (filled circle, 0.2° visual angle) and target (open square, 0.8° visual angle) showed up white against black background. A target hit required that the cursor was inside the target zone for ≥ 15 ms, and once hit, the target immediately reappeared at a new unpredictable position on the screen.

### Instrumented tool.

In all experiments, the thumb and two fingers (index and long finger) of each hand grasped cylindrical handles (diameter = 3.4 cm, length = 3 cm) attached to each side of a rectangular box (12 × 8 × 3.5 cm ^3^). Both handles were equipped with custom-built optometric force transducers located in the box (bandwidth: DC, 120Hz; accuracy better than ± 1.6% within the relevant force ranges). Flags that moved in proportion to the applied longitudinal force (0.05 mm/N) and twist force (0.03 mm/Ncm) gated the intensity of pulsed light (650 nm) transferred through optical fibers to photosensitive diodes. The mean values of the longitudinal and twist forces at the two handles controlled the cursor's position on the screen after filtered at 5 Hz. With no force applied, the cursor was at its center. Horizontal tool movements were measured by a CCD camera [ 49] that tracked the position of a reflex marker located on the box from a direction about perpendicular to the long axis of the tool (resolution better than 0.02 mm). Referenced to that marker, tool rotations were captured by the vertical position of a second marker, located at the tip of a 20-cm long thin balsa rod extending from the box in about the direction of the camera (resolution better than 0.01°). In the fMRI experiments, the camera viewed the markers through a window into the scanning room and via a mirror.

### Experimental series focusing on behavioral measures.

Eight participants (four males and four females; ages: 21–27 y) participated. With their elbows supported on a table, participants sat in front of a vertical computer screen, located ˜40 cm in front of their eyes. Each participant performed four sessions, each requiring 602 target hits. The targets were located in all four quadrants of the screen (see inserts in Figure 1C) and the distance between successive targets was 6.8 ± 1.4° visual angle (mean ± 1 standard deviation [SD]), which corresponded to 2.9 ± 0.6 N change in force applied tangentially to the surfaces of the handles. Two sessions used the right-hand (RH) map and two the left-hand (LH) map. The order of map presentation was for four of the participants LH–RH–RH–LH, and was RH–LH–LH–RH for the remaining four. After each chunk of 43 hits, the participants relaxed for 30 s while they obtained performance feedback as the time (s) they had spent to hit these targets. They rested for 10 min between sessions.

### Neurophysiological experiments.

Eight different people participated in the EMG/TMS experiments (five males and three females; ages: 21–42 y), which were run in a separate experimental series. Out of nine participants initially tested one was excluded because the hand-asymmetry indices indicated dissociation between acting hand for twist and longitudinal forces akin to that observed for two participants in the first experimental series ( Figure 1D and 1E). The participants reclined in a supine position on a firm bed with the screen located about 1 m above the eyes. The back of the head rested on firm foam rubber. An elastic band around the forehead secured the head position with reference to the TMS coil mounted on a stable floor support. The upper arms were parallel to the trunk and a frame, extending above the hips, supported the ulnar sides of the forearms. The wrists were gently strapped to the frame. After the participant had practiced enough to approach plateau performances in hit time with each mapping rule ( Figure 1B), we recorded one continuous run with TMS delivered to the left hemisphere and one to the right hemisphere. For each run and each of the four peripheral targets (see Results), 20 stimulated trials were randomly interspersed amongst 60 control trials without TMS. The order of runs was counterbalanced across participants, who rested for ˜10 min between the runs while the coil was positioned and the stimulus strength adjusted.

Surface EMG was amplified, rectified, and low-pass filtered as previously described [ 22]. TMS was delivered by a MagLite r25 stimulator (Dantec Medical, Skovelund, Denmark) through an angled (18°) figure-eight coil (9-cm wing diameter) positioned optimally for evoking a short-latency response in contralateral hand muscles [ 22]. The stimulus strength was nominally set to 80% of the threshold value obtained in the relaxed participants, which is a preferred intensity for assessing excitability changes in cortico-spinal-motoneuronal pathways during object manipulation [ 22]. Checked after the runs, the strength was 83 ± 9% of the threshold value (mean ± 1 SD across all 16 stimulated hemispheres).

### Functional brain imaging.

Using a 1.5T Philips Gyroscan ACS NT scanner (Philips Medical Systems, Eindhoven, The Netherlands), one fMRI scan of the whole brain was collected every 3.0 s. A gradient-echo sequence provided multi-slice T2*-weighted images (echo time = 50 ms, flip angle = 90° and in-plane resolution = 3.44 × 3.44 mm ^2^ in a 64 by 64 matrix) and an image volume comprised 33 continuous slices of 4.4 mm thickness (no interslice gap). To cope with T1 equilibration effects, five surplus scans were collected before each scanning session. The participants' posture and arm support was the same as in the EMG/TMS experiments. Head movements were minimized using foam pads and an individually molded bite-bar. Headphones reduced scanner noise and participant saw the screen via mirrors attached to the head coil.

Out of 20 people initially tested, sixteen participated (eight males and eight females; ages: 23–37 y). The remaining four had hand-asymmetry indices that indicated dissociation between acting hand for twist and longitudinal forces ( Figure 1D and 1E). Each participant completed four consecutive scanning sessions, each including one bimanual run with the right-hand map and one with the left-hand map. In addition, with the tool clamped to the support frame each session included one run with only the left hand (left-hand map), and one with the right hand (right-hand map). The order of presentation of the four types of runs varied across the four scanning sessions in a manner unpredictable to the participant. Furthermore, we counterbalanced the overall order of runs across participants. In all runs, the distribution of target positions were similar to that in the experiment focusing on behavioral analyses (see Figure 1C), with a mean distance between consecutive targets of 8.8° that corresponded to 3.75 N change in tangential force applied to the handles.

Between scanning sessions, there were 2 min breaks. During an 18-s preparation period preceding each run, a drawing displayed on the screen indicated how to grasp the tool by showing it held by either the right, left, or both hands (cf. Figure 1A). The participants grasped the tool as indicated once the drawing appeared and held it while waiting for the run to commence, triggered by the display instead showing the cursor and the first target. After 36 s of target chasing, the cursor and target extinguished and the participant released the tool and rested while watching a cross hair that appeared in the center of the screen. After 18 s of rest, a new preparation period commenced. Each session begun and ended with an 18-s rest period. The practicing before the scanning sessions took place in a 1:1 model of the MR scanner that included a head-coil, earphones, recorded scanning noise, and bite-bar. In each of four consecutive days occurring 1–3 wk before scanning, subjects performed two consecutive practicing sessions of 16 target-chasing runs (36 s each). A fifth day of training took place 2 or 3 d before scanning. Counterbalanced across the participants, during each of the first 2 d the participants practiced with one or the other of the two maps and performed both bimanually and unimanually. During the 3rd–5th day, the protocol included all four combinations of grasps and maps as during the scanning.

### Data analysis.

A microcomputer-based system digitized and stored force, movement and EMG signals at 400 Hz, 200 Hz, and 1600 Hz, respectively. This system also managed target presentations, assessed target hits, controlled the timing of the magnetic stimuli (TMS), and administrated the fMRI scanning protocol. In statistical assessment of behavioral, EMG, and TMS data the level of probability chosen as significant was *p* < 0.05. When appropriate, before running repeated measures ANOVAs we logarithmically transformed dependent variables to obtain approximately normal distributions and product-moment correlation coefficients were converted to *Z*-scores according to Fisher's transformation. Prior to the correlation analyses used to estimate hand-asymmetry indices, force and movement signals were band-pass filtered (0.2 Hz–4 Hz; ˜12 dB/octave roll-off) to reduce effects of slow tool drifts and of physiological muscle tremor. Statistical significance of obtained correlations was assessed after correction for autocorrelations of the underlying variables [ 50].

For the EMG and TMS experiments, the EMG signals were first transformed to a logarithmic amplitude scale [ 22]. Referenced to the time of appearance of the peripheral target, we then averaged the appropriate time varying EMG, force, torque, and movement signals for each participant and condition (combination of map and peripheral target for non-stimulated trials, and for TMS trials map, peripheral target and stimulated hemisphere). For the “average trials” without TMS we quantified the use of each muscle by the relative change in EMG amplitude from the mean amplitude during a 100-ms baseline epoch commencing when the peripheral target appeared to the point of maximum rate of force change (forces low pass filtered with 20-Hz cut-off frequency). For horizontal and vertical cursor movements this point occurred 0.35 ± 0.04 s and 0.33 ± 0.04 s after target appearance, respectively (mean ± SD for participant means). For “average trials” with TMS, the computer searched each EMG channel for a peak voltage occurring between 15 and 30 ms after the magnetic stimulus. That is, it searched for the peak amplitude of the short-latency component of EMG responses evoked in hand muscles [ 22, 51]. Using the same algorithm, the corresponding background EMG amplitudes were measured from matching non-stimulated “average trials” for computation of the peak:background ratio. This normalization guarded for confounding effects related to changes in the background excitability level of motoneurons and facilitated comparison of effects across different muscles [ 22].

The fMRI data was analyzed using the Statistical Parametric Mapping Software (SPM2, http://www.fil.ion.ucl.ac.uk/spm; Wellcome Department of Imaging Neuroscience, London, United Kingdom). The functional images were resliced, realigned, unwarped to correct for head movements, normalized (linear and non-linear transformation) to the format of the Montréal Neurological Institute (MNI) standard brain (2 × 2 × 2 mm ^3^ voxel size), and smoothed with an isotropic Gaussian kernel of 10 mm (FWHM). High-pass filtering (2.3 mHz) reduced participant-specific drifts in signal and proportional grand mean scaling applied over each scanning session reduced slow global changes in activity. For each participant, we fitted a general linear model to the data (“first level analysis” [ 52]). Eleven different “boxcar” regressors modeled the various functional states of the participants during the scanning. Three regressors represented the preparation periods (left-hand, right-hand, and bimanual grasp). For each type of run, we defined one regressor for the first 6-s period (4 regressors) and one regressor for the remaining 30 s of the runs (4 regressors). Because our focus here was on neural correlates of steady-state adapted performance, the regressors pertaining to the preparation period and the first 6-s period of the bimanual runs as well as regressors pertaining to unimanual performances represented states of no interest. The regressors were convolved with the standard canonical hemodynamic response function and the general linear model provided the relevant contrast images for each of the 16 participants. We then entered those into a random effects group analysis (“second level analysis” [ 52]) and used one-sample *t*-tests to label regions of activity changes. When contrasting BOLD signals obtained with the left-hand map with those obtained with the right-hand map, and vice versa (see Table 1), we used a *p*-value threshold of 0.01 that was corrected for multiple comparisons at the level of single voxels with the false discovery rate algorithm [ 53]. Based on functional images for each experimental condition and participant, we obtained the comparisons in the conjunction analysis by means of multiple-regression in SPM2. The conjunction was defined as previously described [ 54] and the FWE algorithm was used to correct for multiple comparisons [ 53], using a corrected *p*-value threshold of 0.01. The anatomical localization of local maxima (and clusters) was assessed by reference to the Talairach stereotactic atlas [ 55] after appropriate coordinate transformation ( http://www.mrc-cbu.cam.ac.uk/Imaging) and for the cerebellum by reference to the Schmahmann atlas [ 56]. Minimum required spatial cluster extent was 20 voxels and local maxima had to be separated by ≥ 10 mm. Visualization of significant effects on brain templates and plots of effects in histograms were done using in-house developed software ( *DataZ*).

## Abbreviations

1DI: first dorsal interosseous
ANOVA: analysis of variance
APB: abductor pollicis brevis
BA: Brodmann area
BOLD: blood oxygenation level-dependent
ECR: extensor carpi radialis
EMG: electromyogram
FCU: flexor carpi ulnaris
fMRI: functional magnetic resonance imaging
FWE: family-wise error
LH: left hand
Lo: cerebellar lobule
RH: right hand
SD: standard deviation
TMS: transcranial magnetic brain stimulation
